# Inferring task-related networks using independent component analysis in magnetoencephalography

**DOI:** 10.1016/j.neuroimage.2012.04.046

**Published:** 2012-08-01

**Authors:** H. Luckhoo, J.R. Hale, M.G. Stokes, A.C. Nobre, P.G. Morris, M.J. Brookes, M.W. Woolrich

**Affiliations:** a Oxford Centre for Human Brain Activity, University of Oxford, Warneford Hospital, Oxford, UK; b Sir Peter Mansfield Magnetic Resonance Centre, School of Physics and Astronomy, University of Nottingham, University Park, Nottingham, UK; c Centre for Doctoral Training in Healthcare Innovation, Institute of Biomedical Engineering, Department of Engineering Science, University of Oxford, UK; d FMRIB Centre, University of Oxford, Oxford, UK

**Keywords:** MEG, Working memory, Independent component analysis, General linear model, Hippocampus, Neural oscillations

## Abstract

A novel framework for analysing task-positive data in magnetoencephalography (MEG) is presented that can identify task-related networks. Techniques that combine beamforming, the Hilbert transform and temporal independent component analysis (ICA) have recently been applied to resting-state MEG data and have been shown to extract resting-state networks similar to those found in fMRI. Here we extend this approach in two ways. First, we systematically investigate optimisation of time-frequency windows for connectivity measurement. This is achieved by estimating the distribution of functional connectivity scores between nodes of known resting-state networks and contrasting it with a distribution of artefactual scores that are entirely due to spatial leakage caused by the inverse problem. We find that functional connectivity, both in the resting-state and during a *cognitive* task, is best estimated via correlations in the oscillatory envelope in the 8–20 Hz frequency range, temporally down-sampled with windows of 1–4 s. Second, we combine ICA with the general linear model (GLM) to incorporate knowledge of task structure into our connectivity analysis. The combination of ICA with the GLM helps overcome problems of these techniques when used independently: namely, the interpretation and separation of interesting independent components from those that represent noise in ICA and the correction for multiple comparisons when applying the GLM. We demonstrate the approach on a *2-back* working memory task and show that this novel analysis framework is able to elucidate the functional networks involved in the task beyond that which is achieved using the GLM alone. We find evidence of localised task-related activity in the area of the hippocampus, which is difficult to detect reliably using standard methods. Task-positive ICA, coupled with the GLM, has the potential to be a powerful tool in the analysis of MEG data.

## Highlights

► We outline two methods for detecting functional networks in task positive MEG data. ► First is an analysis to find the optimum time-frequency window for FC detection. ► The second combines independent component analysis with the general linear model. ► Methods are applied to resting state and working memory task data. ► ICA/GLM reduces multiple comparisons and localises hippocampi.

## Introduction

Current theories of brain function postulate that a relatively small number of spatially distributed networks are involved in many cognitive functions. These networks are recruited through spontaneous synchronisation of neural oscillations across different, spatially separate regions (Uhlhaas and Singer, 2010). This synchrony is not attributed to the physical connections between the regions alone but has an underlying functional component. Methods for quantifying this functional connectivity are critical to furthering the general understanding of how the brain carries out cognitive tasks.

One such method is independent component analysis (ICA). ICA is a widely used blind source separation technique that decomposes a mixture of signals into a set of statistically independent components (Bell and Sejnowski, 1995). It has been used to analyse both task-based fMRI data (McKeown et al., 1998) and resting-state fMRI data (Beckmann et al., 2005; Smith et al., 2009). In addition, ICA has been widely applied in magnetoencephalography (MEG) and electroencephalography (EEG), although primarily as a means of removing artefacts and denoising sensor space data (e.g. Jung et al., 2001). More recently, ICA has been applied to MEG data in source space in order to extract resting-state networks (Brookes et al., 2011c). Eight networks, including the default mode and lateral frontoparietal networks, were found using temporal ICA on the oscillatory amplitude envelope, showing that ICA is effective at extracting functionally connected networks in resting-state MEG data. Brookes et al. reported that their best measurements of functional networks were found by analysing the low-frequency fluctuations in the oscillatory envelope of the beta band (at a time scale of ~ 1 s); however their justification for this is based on a consideration of connectivity between the left and right motor cortices alone (Brookes et al., 2011b) and there was no systematic investigation of optimal time-frequency windows for robust functional connectivity measurement.

Task-positive MEG data can be analysed using either mass univariate General Linear Modelling (GLM) (Brookes et al., 2004; Friston et al., 1996) or pseudo-T-statistical approaches (Vrba and Robinson, 2001) to identify significant task-related changes. However, one of the limitations of applying any mass univariate statistical test in individual and group level analyses is that corrections must be applied for multiple comparisons (Nichols and Hayasaka, 2003; Woolrich et al., 2009). This issue is particularly challenging in MEG where tests can be carried out over space, time, frequency and subject. GLM multiple comparisons corrections have been adapted for specific use in MEG; including methods to capture the inhomogeneous spatial smoothness of source space MEG data at the individual level (Barnes and Hillebrand, 2003), and group level permutation methods (Singh et al., 2003). However, these corrections can become prohibitively severe as more statistical tests are carried out (i.e. with more voxels or subjects).

In this paper, we present two methodological developments. The first is a general, non-parametric framework for estimating the optimum time-scales and frequency windows over which functional connectivity can be robustly detected in MEG data. This is achieved by estimating the distribution of functional connectivity scores between nodes of known resting-state networks and contrasting it with a distribution of artefactual scores that are entirely due to spatial leakage caused by the ill-posed inverse problem (Schoffelen and Gross, 2009). The optimisation analysis is applied to resting-state and task-positive data, and is used to set the frequency bands and time-scale used in the subsequent combined ICA/GLM analyses.

A second methodological development extends the ICA approach previously developed to extract resting-state networks from MEG data (Brookes et al., 2011c) by combining it with the GLM to allow for inference of task-related activity on functional networks. In the first stage of the analysis temporal ICA is applied to oscillatory amplitude envelopes in source space. The resulting independent components are assumed to represent task-related networks of neural activity, task-irrelevant functional networks and non-neural artefacts (in the case of ideal un-mixing). The GLM is then applied to each independent component time course to identify the functional networks most closely associated with the experimental task. This use of ICA in combination with the GLM greatly reduces the number of statistical tests carried out, thus substantially ameliorating the multiple comparisons problem. Whilst the use of GLM inference on the output from ICA has been carried out previously on fMRI data (Beckmann et al., 2006), this is the first time it has been used in the context of an ICA method that can independently identify functional networks in electrophysiological data (Brookes et al., 2011c).

The method is applied to a working memory task. Working memory (WM) is the system for temporary storage of information for use in complex tasks (Baddeley, 1992). As such, it is recruited for a wide range of functions and abnormal working memory is associated with various neurological pathologies including Alzheimer's disease and schizophrenia (Baddeley, 1992; Egan et al., 2001). Previously, Brookes et al. (2011a) investigated working memory using MEG and reported a strong theta band (4–8 Hz) event-related oscillatory power increase due to WM in the frontal regions and a corresponding visual theta oscillatory power decrease. A decrease in oscillatory power in the high beta/low gamma band (20–40 Hz) in the frontal and posterior parietal areas was also reported. It is this complex recruitment of functional networks that makes the task ideal for testing the task-positive ICA methodology as the *2-back* data is extremely rich in both temporal and spatial functional information.

## Materials and methods

### Participants

Twelve healthy volunteers participated in a *2-back* working memory MEG study. The cohort comprised 6 males and 6 females with a mean age of 25 years and standard error of 1 year. The study was approved by the University of Nottingham Medical School Research Ethics Committee.

### Experimental paradigm

**Fig. 1:**
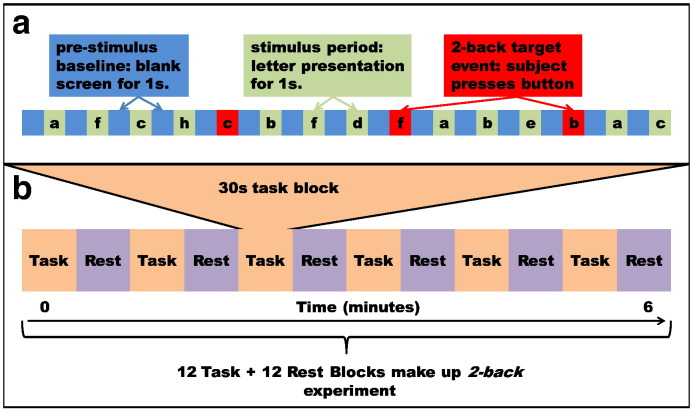
Schematic of the *2-back* paradigm. (a). The stimulus train: a 1 s blank screen preceded a 1 s letter presentation to make a single trial. Whenever the current letter matched that of the one two trials earlier, the subject pressed a button. 15 trials made up a 30 s task block. (b). Each task block was followed by a 30 s rest block. 12 task blocks, each followed by a rest block, made up a single experiment, lasting 12 min.

We acquired MEG data for each participant both in the resting-state (300 s during which the subject was asked to lie still with their eyes open and fixate on a cross central to their field of vision) and also during a *2-back* working memory task. The *2-back* paradigm involved presenting the subject with a series of stimuli; in this instance lower case letters. The subject was asked to respond (via a button press with the index finger of the right hand) if the current stimulus matched that presented two stimuli earlier (referred to as a target). Here, a 1 s baseline period, consisting of a blank screen, preceded a 1 s letter presentation; therefore each trial lasted a total of 2 s. 15 trials were combined to make a single 30 s task block. Each task block was followed by a 30 s rest block during which the fixation cross was shown. A total of 12 task blocks, each followed by a rest block, were collected for each subject, giving a total of 12 min of *2-back* data. The 300 s resting-state recording preceded the *2-back* task. Fig. 1 shows a schematic of the *2-back* experiment. The task was implemented in *PsychoPy* (www.psychopy.org) and visual presentation was achieved via projection through a waveguide in the magnetically shielded room onto a screen placed ~ 40 cm in front of the subject.

### Data acquisition

MEG data were acquired using a 275 channel CTF whole-head system (MISL, Coquitlam, Canada). The data were acquired at a sampling frequency of 600 Hz and synthetic 3rd order gradiometer correction was applied to reduce external interference. Head localisation within the MEG helmet was achieved using three electromagnetic head position indicator (HPI) coils (placed at three fiducial points: nasion, left and right pre-auricular points). By periodically energising these coils the head position within the MEG sensor array was identified. Prior to data acquisition, the HPI coil locations and the subject's head shape were digitised using a Polhemus Isotrack system. Structural MR images for each subject were acquired using a Philips Achieva 3T MRI system (MPRAGE; 1 mm isotropic resolution, 256 × 256 × 160 matrix, TR = 8.1 ms, TE = 3.7 ms, TI = 960 ms, shot interval = 3 s, flip angle = 8° and SENSE factor 2). The locations of the MEG sensors were co-registered to the brain anatomy by matching the digitised head surface to the head surface extracted from the anatomical image.

### Data analysis

Basic artefact rejection was applied by visually inspecting each task and rest block pair and removing block pairs containing obvious artefacts (such as muscle artefacts which exhibit a distinct frequency structure) or unusually high variance. The remaining, uncorrupted blocks from each subject were used in all subsequent analyses. The individual registration of each subject's head shape to their structural MRI was carried out using *SPM8* (www.fil.ion.ucl.ac.uk/spm). The forward model was estimated using a single homogenous shell model of the head shape of each subject (Mosher et al., 1999). MEG data were then bandpass filtered into three frequency bands (*theta*: 4–8 Hz; *alpha/low beta*: 8–20 Hz; *high beta/low gamma*: 20–40 Hz). These bands were chosen from the *optimisation of down-sampling* analysis.

In order to perform connectivity analysis in brain space, we required a method to map the sensor space MEG data into source space. Exact projections do not exist because the MEG inverse problem is ill-posed but a range of approximate source reconstruction techniques is available. Here, we projected the MEG data into source space using a linearly constrained minimum variance beamformer (Robinson and Vrba, 1999; Van Veen et al., 1997; Woolrich et al., 2011). A beamformer is an adaptive spatial filter in which a time series of electrical activity at a location of interest in source space is estimated as a weighted sum of MEG sensor measurements. Projections require an estimate of covariance between all sensor pairs and a forward model comprising the modelled fields that would be measured at each MEG sensor in response to a unit current at the location of interest with known orientation (in this case the forward model included a single shell BEM conductor model combined with Maxwell's equations (Mosher et al., 1999)). Here, the data covariance matrix was regularised according to *C*_*reg*_ *= C* + *μI* where *μ* equals 4 times the minimum eigenvalue of the unregularised data covariance matrix, *C*. Regularisation in this way acts to spatially smooth the beamformed data and increase the temporal signal-to-noise ratio. Beamformer weights are designed to have unity pass band for the location (and source current orientation) of interest whilst minimising signal variance originating from all other sources. In this implementation, source orientation was estimated as that which exhibited the maximum signal-to-noise ratio, computed using the method developed by Sekihara et al. (2001).

For the *2-back* data, the beamformer was used to provide a source space estimate of the neural activity time series at every vertex of a regular 6 mm grid spanning the entire brain, as well as in both eyeballs (MNI coordinates: ± 34 56–38 mm). For the *optimisation of down-sampling* analysis, beamformer estimated time series were constructed at a set of 12 specific locations (described in Table 1). The oscillatory amplitude envelopes of the reconstructed (band filtered) time series were estimated via computation of the absolute value of the analytic signal, which was found using a *Hilbert transform*. These envelopes yielded an estimate of instantaneous signal amplitude. The envelope time series for every voxel were down-sampled by dividing each voxel's envelope time series into equal windows of duration Δ and calculating the mean value of the envelope within each window. This process was equivalent to low-pass filtering and down-sampling the amplitude envelope.

#### Optimisation of down-sampling

Down-sampling of the amplitude envelope has been shown to enhance functional connectivity (FC) measures between regions that are known to be connected, such as the left and right motor cortices (Brookes et al., 2011b). The windowed down-sampling effectively low pass filters the data, focussing on low frequency amplitude fluctuations that are thought to be direct manifestations of electrophysiological functional connectivity (Brookes et al., 2011b; de Pasquale et al., 2010; Liu et al., 2010; Mantini et al., 2007). However, whilst down-sampling may optimise the temporal scale of the data for FC metrics such as correlation, it also necessarily increases the absolute correlation between unconnected pairs due to the reduction in sample count. This is because the standard error on the correlation coefficient is proportional to 1/√N (where N is the number of observations). However, because we are using the absolute value of the correlation coefficient, this causes the mean of the correlation coefficient under the null hypothesis also to be proportional to 1/√N. Brookes et al. (2011b) used Monte Carlo simulations to show that the optimum separation between genuinely connected and unconnected voxel pairs in resting-state data was achieved for window lengths of the order of 1 s. However, this result was found through a consideration of the sensorimotor network during the resting-state only.

In this study, we developed a more general approach. For each subject in the *2-back* cohort, the beamformer was used to estimate the activity at voxels in the default mode network (DMN), lateral frontoparietal network (LFPN), sensorimotor network and hippocampi over a range of frequency bands, between 0 and 150 Hz in non-overlapping 4 Hz windows. The coordinates of these nodes are listed in Table 1. From these 12 nodes, we estimated 10 pair-wise functional connectivity scores (Pearson correlation between down-sampled oscillatory amplitude envelopes) for each subject, which, when combined, gave us a population of FC values for voxel pairs thought to be genuinely connected. The node pairs used were the following: 1.) *anterior* to *posterior left LFPN*; 2.) *anterior* to *posterior right LFPN*; 3.) *left* to *right motor cortices*; 4.) *left* to *right hippocampi*; 5.) *left* to *right parietal DMN*; 6*.*) *posterior cingulate cortex* to *left parietal DMN*; 7.) *posterior cingulate cortex* to *right parietal DMN*; 8.) *left parietal DMN* to *anterior* DMN; 9.) *right parietal DMN* to *anterior DMN*; 10.) *posterior cingulate cortex* to *anterior DMN*. In order to estimate the distribution of artificial pair-wise correlation scores, we projected uncorrelated (and bandpass filtered) Gaussian noise through the same beamformer weights used to estimate the sources at the genuinely connected nodes in Table 1. 50 instantiations per set of beamformer weights were generated to populate the unconnected distribution. These simulated time courses had no underlying connectivity and so any FC was spurious and entirely due to beamformer cross-talk. Connected and unconnected pair-wise correlation populations were estimated for the *2-back* task and rest blocks, the *2-back* task blocks only and resting-state data. Note that in using the *2-back* task data to assess the choice of down-sampling window, we were not using any information about the stimulus timings. The oscillatory amplitude envelopes were estimated and the signals down-sampled for a range of window lengths (of duration Δ = 0, 0.1, 0.25, 0.5, 0.7, 1, 1.5, 2, 3, 4, 5, 7, 10 s). Absolute correlation scores (as negative and positive envelope correlations are equally indicative of FC) were calculated for each frequency band for all values of window length Δ.

The un-normalised Fisher transformation (Eq. (1)) was applied to the data to map the limited range (0 to 1) of absolute Pearson correlation values into an infinite space. A two parameter gamma distribution was then fitted separately to the connected and unconnected populations of pair-wise absolute correlation values. The gamma distribution was ideal because it inherently matched the key properties of the pair-wise absolute correlation distributions: a hard limit at zero, the bulk of the distribution clustered at low positive values but with a tail extending to higher values (see Figs. 3(a, b, c)).

[1] $$z=\frac{1}{2}\ln\frac{1+r}{1-r}$$

For each frequency band and down-sampling window, we estimated the Fisher transformed absolute correlation that corresponded to the 95% threshold using the gamma fit to the unconnected pairs' correlation values. If this value of correlation was used as a threshold, a false positive detection rate of 5% would be expected for classifying unconnected pairs as exhibiting FC. Using the gamma fit to the connected pairs' correlation values, we estimated the equivalent true positive detection probability for this correlation threshold, and used that as a metric for how well the connected and unconnected populations were separated by the down-sampling process. The optimum value of Δ maximised this true positive detection probability in the frequency band of interest.

#### Independent component analysis and general linear model

**Fig. 2:**
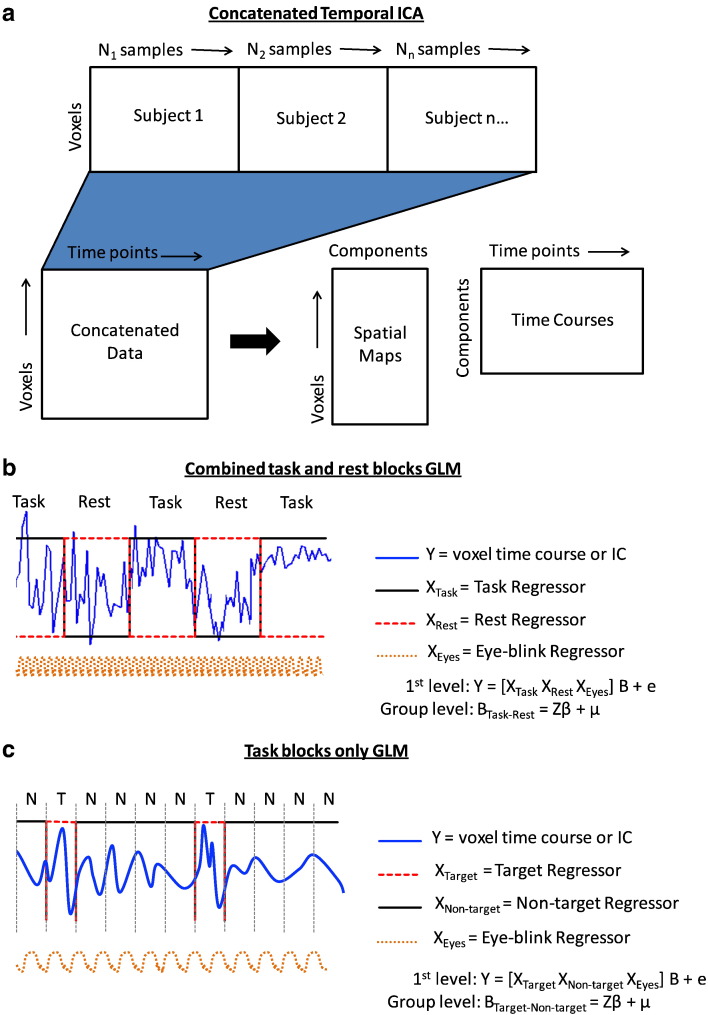
Decomposition of temporally concatenated, multi-subject data using temporal ICA and subsequent testing using the mixed-effects GLM. (a). A schematic of the concatenation of subjects followed by ICA decomposition into its spatial maps (or mixing matrix) and independent time courses. (b). The *combined task and rest* mixed-effects GLM, testing for differences between the task blocks and rest blocks. (c). The *task blocks only* mixed-effects GLM, testing for differences between the target (T) and non-target (N) trials. Both GLMs include the average eye-blink activity for each subject as a nuisance regressor in the 1st level, ensuring that any statistical inferences are orthogonal to eye-blink activity.

The down-sampled amplitude envelopes for the *2-back* data, computed for all source space voxels (6 mm resolution) and all subjects, were temporally concatenated across subjects. Temporal ICA was then performed using the ICASSO algorithm (Himberg and Hyvarinen, 2003). The data were reduced to 20 dimensions using Principle Component Analysis (PCA) and then separated into 20 temporally independent time series (schematically shown in Fig. 2(a)). The ICASSO algorithm carried out 30 iterations of *fastICA* (Hyvärinen, 1999) before clustering the results. Implementations of ICA are based on maximising measures of non-Gaussianity in a high dimensional space through optimisation methods. However, such methods are prone to finding different local maxima depending on the initialisation. ICASSO was implemented to overcome the issues due to random initialisations in the ICA.

Two separate independent component analyses were carried out for each frequency band. In the first, the concatenated data included both the 30 s task blocks and the 30 s rest blocks (henceforth referred to as the *combined task and rest* analysis). In the second analysis, only the task blocks were used (henceforth referred to as the *task block only* analysis).

We included the oscillatory envelope time series of both eyeballs, concatenated over subjects, in the data input into each ICA decomposition. By including these time series, the ICA algorithm was more likely to find a single component that corresponded to the contamination due to eye-blinks.

Each temporal ICA yielded 20 independent time courses. These time courses were each converted into a correlation map by estimating the Pearson correlation coefficient between each independent time course and the down-sampled amplitude envelope time course, concatenated across subjects, associated with each voxel. This gave a correlation map for each independent component found by the ICA. Correlation maps have similar spatial topographies to the spatial maps derived from the ICA mixing matrix; however correlation maps can both be more intuitively thresholded and equated to correlation maps from seed-based connectivity analyses (Brookes et al., 2011a, 2011b, 2011c; de Pasquale et al., 2010).

Alongside the ICA, a general linear model (GLM) (Brookes et al., 2004; Friston et al., 1996; Woolrich et al., 2009) was implemented. The GLM assumes that a signal can be modelled as a linear combination of known regressors and a normally distributed error. With the GLM framework, it is possible to infer whether there are statistically significant oscillatory amplitude differences in the task blocks compared with the rest blocks. In addition, it is possible to see if there is a significant difference between non-target trials and target trials. A GLM was applied to the down-sampled envelopes of the task and rest blocks for each subject, yielding t-statistical maps of regions where there was a significant difference between the task and rest blocks. The average eye-blink envelope time course (taken as the average of the envelopes of each beamformed eyeball time course) was included as a nuisance regressor in the GLM to prevent electrical activity from eye-blinks confounding the GLM statistics. A second, group level, mixed-effects GLM was used to test for inter-subject consistency in the contrast of parameter estimates (COPE) of the task and rest regressors. A schematic of the *combined task and rest* GLM is shown in Fig. 2(b).

A mixed-effects GLM was also applied to the task blocks alone, looking for changes between the *2-back* target trials and the non-target trials, first at the individual and then group level (Fig. 2(c)); in this case the COPE of the target and non-target regressors was passed to the group stage. Again, the average eye-blink envelope was included as a nuisance regressor. Statistical maps for both the *combined task and rest* and *task blocks only* GLM analyses were corrected for multiple comparisons using false discovery rate methods (FDR), as implemented in *FSL* (http://www.fmrib.ox.ac.uk/fsl/).

A mixed-effects GLM was also applied to the independent component time courses found by the *combined task and rest* ICA and the *task block only* ICA. The mixed-effects GLM was implemented by splitting the concatenated ICs back into individual subject time courses and then proceeding to use a two level GLM, the first level including the average eye-blink envelope as a nuisance regressor. The GLM analyses provided a reference for comparison and validation for the ICA correlation maps. In addition, they provided a means to discriminate task-related independent components from those that characterise noise. Note that concatenating the task blocks will produce time courses with discontinuities. However, the presence of discontinuities does not affect the ICA as its results are independent of the data ordering; and the GLM on the IC time courses remains valid as the selection and concatenation of the task block only data can be expressed as a linear matrix operation applied identically to both the data and to the design matrix. When applying the GLM to the independent time courses, a multi-step-up test (Eq. (2)) was used to correct for multiple comparisons (Nichols and Hayasaka, 2003). The ICs were ranked by their mixed-effects p-value, from least to most significant. In step one, the least significant component was tested for significance against a threshold (in this case p = 0.05). If it proved to be non-significant, the next least significant p-value was selected (step two) and multiplied by a scaling factor equal to the step number. If it proved to be non-significant, the next component was tested until one was found to be significant. All remaining (more significant) components were then classed as above threshold. A step-up correction was used as it is equivalent to the FDR corrections applied to the traditional GLM analyses (Nichols and Hayasaka, 2003).

(2) $$\begin{array}{l} \text{i}{\text{f p}}_{\text{i}}\text{> }\frac{\text{α i}}{\text{I}}\text{ then test next component }\left(\text{increment i}\right)\\ {\text{if p}}_{\text{i}}\leq \frac{\text{α i}}{\text{I}}\text{ then classify components i through I as significant}\text{.} \end{array}$$

## Results

### Optimisation of the down-sampling window

**Fig. 3:**
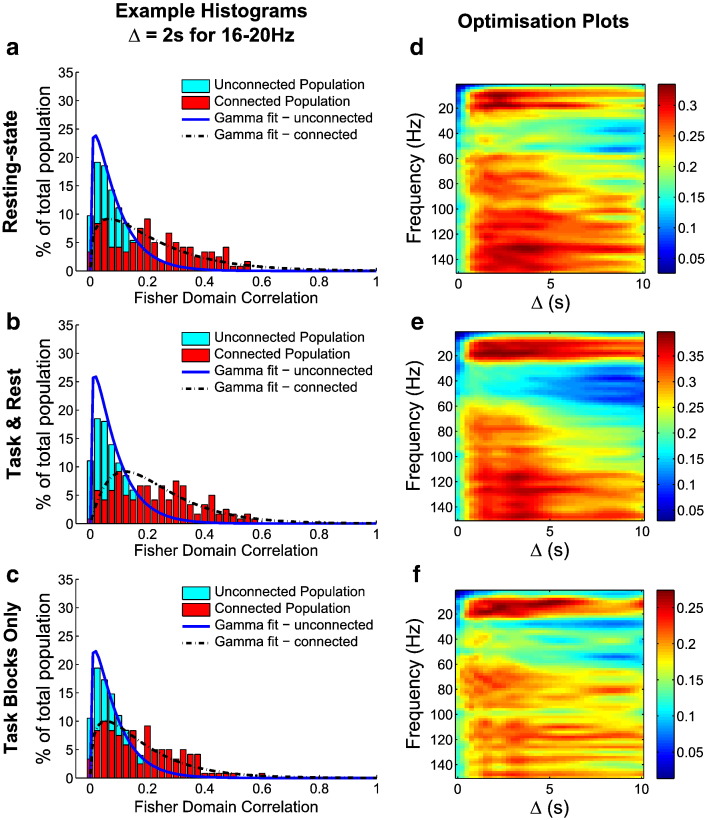
Results of the analysis to identify the optimum down-sampling window length, Δ. The analysis has been performed on the resting-state data (a, d), the *2-back task and rest blocks* (b, e) and the *2-back task blocks only* (c, f). (a, b, c). The normalised histograms of the Fisher transformed pair-wise correlation values for 10 voxel pairs (known to be functionally connected, listed in Table 1) across 12 subjects is shown in red. For each set of beamformer weights, 50 unconnected time courses were simulated, giving an equivalent unconnected population whose histogram is shown in cyan. (d, e, f). The probability of true positive detection of functional connectivity (estimated at the false positive probability of 5%) for a range of frequency bands and down-sampling windows.

Fig. 3 shows the findings of the investigation into the effect of the down-sampling process and the optimum value of Δ for identifying genuine functional connectivity. The histograms for the connected and unconnected populations were well represented by gamma distributions (Figs. 3(a, b, c)) and the probability of true positive detection (assessed at a false positive rate of 5%) identified a clear optimum down-sampling window in both the *2-back* and resting-state data as shown in Figs. 3(d, e, f). It should be noted that in real data, correlations between these node–node pairings of known networks were significantly higher than equivalent correlations derived from simulated data. This was the case even when beamformer cross-talk was taken into account. This is therefore in agreement with previous work, showing that electrophysiological signals correlate across nodes of previously well-characterised ‘haemodynamic’ networks, including the lateral frontoparietal networks, the default mode network, the motor network and bilateral hippocampi. The 8–20 Hz range (alpha and low beta bands) was found to be optimal for detecting functional connectivity and this also agrees with previous work (Brookes et al., 2011b; Liu et al., 2010; Mantini et al., 2007). In the very low frequency bands, > 4 Hz, and in the 30–60 Hz band, the probability of genuinely identifying functional connectivity was lowest. Interestingly, in the high gamma range, 70–150 Hz, we found evidence of strongly detectable envelope correlations.

Across all frequency bands, the down-sampling greatly improved separation between the connected and unconnected populations for windows up to 1 s ≤ Δ ≤ 4 s. The optimum Δ varied depending on which node pair is considered but, in general, 1 s ≤ Δ ≤ 4 s was a good whole-brain estimate of the optimum value of window length Δ. Within this window, the probability of successfully detecting genuine functional connectivity was maximal.

Based on these optimisation results the window length, Δ, for the *2-back* ICA was set at 0.5 s for the three frequency bands considered. This approached the optimal operating point for robustly detecting functional connectivity, whilst at the same time allowing the analysis to retain within-trial sensitivity. It is worth noting that the optimisation analysis on the concatenated *task blocks only* has shown that setting Δ to 0.5 s is sensible, even though in the *task block only* analysis, due to the limited block length of 30 s, low frequency oscillatory envelope oscillations will be harder to estimate robustly.

The optimisation of down-sampling analysis showed that optimum detection of functional connectivity was found in the 8–20 Hz band. However, in previous work, it was observed that the theta and high beta/low gamma bands were closely associated with working memory (Axmacher et al., 2010; Brookes et al., 2011a). Therefore, the theta band (4–8 Hz) and the high beta/low gamma band (20–40 Hz) were also included in subsequent analyses.

### Results of a GLM alone

**Fig. 4:**
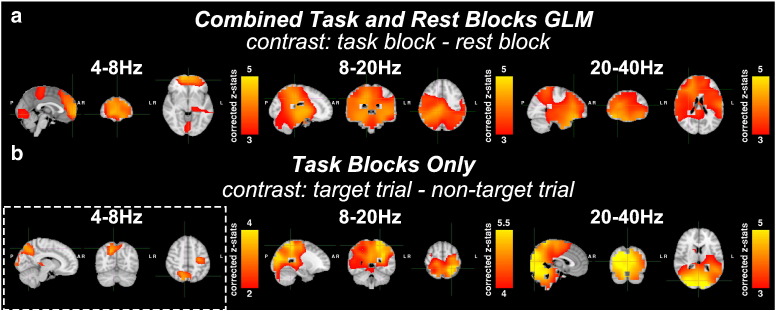
Results of mixed-effects GLM analyses on the down-sampled oscillatory envelopes at each voxel. Maps of corrected z-statistics are shown in radiological view (axial, coronal and sagittal views from left to right). Z-statistics were corrected using false discovery rate (FDR) methods in *FSL* and subsequent z-statistical maps thresholded between 3 and 5 for visualisation, except the *task blocks only* 4–8 Hz (2 < z < 4) and 8–20 Hz (4 < z < 5.5) maps. An FDR of 0.05 was used for all the GLMs except the 4–8 Hz *task blocks only* analysis (in the white dashed box) where a FDR of 0.2 was used as no activity survived corrections at a FDR of 0.05. (a). Result of *combined task and rest* GLMs testing for differences between the 30 s task and rest blocks for the 4–8 Hz, 8–20 Hz and 20–40 Hz bands. (b) Result of *task blocks only* GLM testing for changes between the trials where a target was presented against all other non-target trials. In all GLMs, the average eye-blink time course was included as a nuisance regressor and hence all statistical inferences are orthogonal to eye-blink activity.

Fig. 4 shows the results of the traditional GLM analyses: z-statistical maps of the group level GLM regression parameters are shown. Fig. 4(a) shows the corrected z-statistics for the contrast of parameter estimates between regressors for the task blocks and rest blocks (thresholded between 3 and 5). Maps were produced for the 4–8 Hz, 8–20 Hz and 20–40 Hz bands. For the 4–8 Hz band, significant activity was seen in the frontal, visual and motor regions. In the 8–20 Hz band, significant activity was found in the parietal and superior temporal lobes but this activity was not well localised to any specific regions. In the 20–40 Hz band, significant task-related activity was seen in the parietal, frontal and temporal regions, possibly resembling the default mode and salience networks.

Fig. 4(b) shows the z-statistical maps for a GLM analysis confined to the task blocks of the down-sampled envelope (thresholded between 2 < z < 4 for the 4–8 Hz band, 4 < z < 5.5 for the 8–20 Hz band and 3 < z < 5 for the 20–40 Hz band). In the 4–8 Hz band, activations were observed in the visual cortex, medial superior parietal lobule and the left motor cortex. For the 4–8 Hz *task blocks only* GLM, the FDR threshold was changed from 0.05 to 0.2. For a FDR correction of 0.05, there was no significant activity. The correction threshold was changed to allow visualisation of the theta band activity. In the 8–20 Hz band, there were significant changes (p_corrected_ < 0.05 at FDR threshold of 0.05) in oscillatory amplitude observed in the parietal regions. In the 20–40 Hz band, significant amplitude changes were found in the parietal lobes, visual areas and cerebellum.

### Results of a combined ICA/GLM

#### ICA applied to *combined task and rest blocks*

**Fig. 5:**
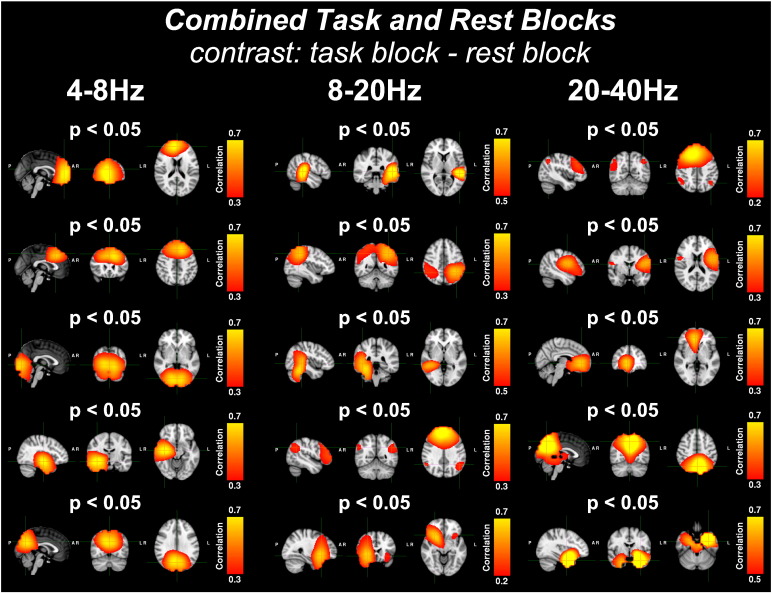
Shows the correlation maps generated from ICAs applied to the *combined task and rest blocks* for the down-sampled envelopes of three frequency bands (4–8 Hz, 8–20 Hz, 20–40 Hz), concatenated over subjects. Correlation maps, presented in radiological view (axial, coronal and sagittal slices from left to right), show the correlation between each temporally independent component and the concatenated down-sampled envelopes of each voxel. Variable correlation thresholds were used to aid visualisation and are shown on each map's colour bar. Independent time courses were tested using a mixed-effects GLM, which identified components that showed strong changes between the task and rest blocks. P-values were corrected for multiple comparisons using a multi-step-up test (Nichols and Hayasaka, 2003). In all GLMs, the average eye-blink time course was included as a nuisance regressor and hence all statistical inferences are orthogonal to eye-blink activity. For each frequency band, five correlation maps are presented for components whose corrected p-values were found to be significant (p < 0.05). From top to bottom, maps correspond to: 4–8 Hz: frontal area, separate frontal area, visual cortex, right hippocampus, and superior parietal lobule; 8–20 Hz: left superior temporal gyrus, bilateral superior parietal lobule, right superior temporal gyrus, default mode network, and bilateral insula; 20–40 Hz: default mode network, somatosensory network, cingulate, medial superior parietal lobule, and bilateral hippocampi.

Fig. 5 shows the results of an ICA of the *combined task and rest blocks* of the down-sampled envelope in the 4–8 Hz, 8–20 Hz, 20–40 Hz frequency bands, concatenated across subjects. A mixed-effects GLM was used to estimate which independent time courses demonstrated significant task-related changes in oscillatory amplitude between the task and rest blocks. Significant (p_corrected_ < 0.05) correlation maps for five components are shown for each band (note that for the *combined task and rest* analyses, more than five components were significant in each band but only five have been presented for clarity). The correlation maps were windowed with a variable correlation threshold to aid visualisation of the different networks.

##### Theta band (4–8 Hz)

For the theta band (4–8 Hz), the correlation maps localised significant activity in the frontal lobe, consistent with the *combined task and rest block* GLM analysis shown in Fig. 4(a). However, the ICA split the frontal activations into two distinct components. In addition, the ICA identified significant theta band oscillatory amplitude changes in the task blocks relative to the rest blocks in the visual cortex and medial superior parietal lobule (SPL), again consistent with the z-statistical maps derived from a traditional GLM analysis. Finally, a component corresponding to activity in the right hippocampus was identified.

##### Alpha/low beta band (8–20 Hz)

In the alpha and low beta band (8–20 Hz), two significant components were extracted that corresponded to the left and right superior temporal gyri. An additional significant component was found that corresponded to bilateral SPL activity. Finally, two significant components were identified that resembled the default mode network and bilateral insula.

##### High beta/low gamma band (20–40 Hz)

In the high beta/low gamma band (20–40 Hz), the most significant component resembled the default mode network. Components corresponding to the somatosensory network, cingulate, medial SPL and bilateral hippocampi were also found.

#### ICA/GLM confined to *task blocks only*

**Fig. 6:**
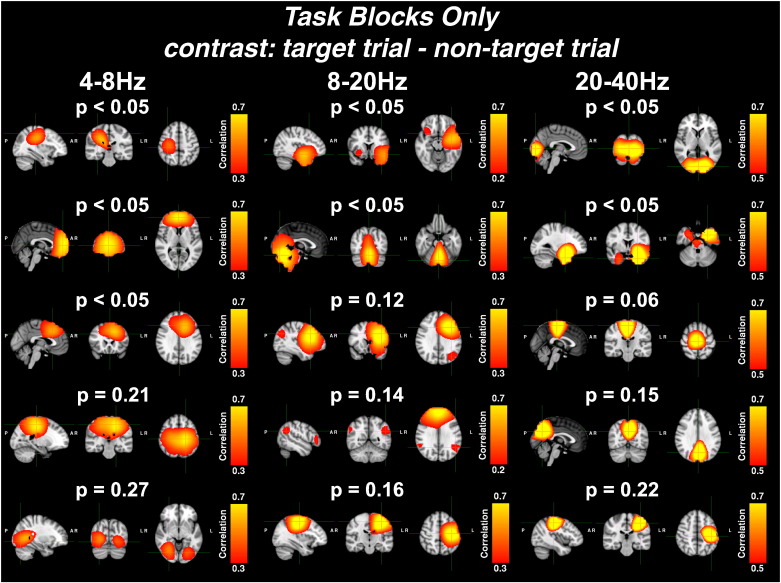
Shows the correlation maps generated from ICAs applied to the *task blocks only* for the down-sampled envelopes of three frequency bands (4–8 Hz, 8–20 Hz, 20–40 Hz), concatenated over subjects. Correlation maps, presented in radiological view (axial, coronal and sagittal slices from left to right), show the correlation between each temporally independent component and the concatenated down-sampled envelopes of each voxel. Variable correlation thresholds were used to aid visualisation and are shown on each map's colour bar. Independent time courses were tested using a mixed-effects GLM which identified components that show strong changes between target and non-target trials. P-values were corrected for multiple comparisons using a multi-step-up test (Nichols and Hayasaka, 2003). In all GLMs, the average eye-blink time course was included as a nuisance regressor and hence all statistical inferences were orthogonal to eye-blink activity. For each frequency band, five correlation maps are presented for components whose corrected p-values are significant (p < 0.05) or whose spatial localisation is functionally interesting. From top to bottom, maps correspond to: 4–8 Hz: right motor cortex, frontal areas, separate frontal areas, parietal cortex, and lateral visual areas; 8–20 Hz: bilateral insula, medial cerebellum, left lateral frontoparietal network, default mode network, and left motor cortex; 20–40 Hz: visual cortex, bilateral hippocampi, premotor areas, medial superior parietal lobule, and left motor cortex.

Fig. 6 shows the results of the independent component analysis confined to the *task blocks only* of the *2-back* experiment. Again a mixed-effects GLM was used to estimate which independent time courses demonstrated significant task-related changes in oscillatory amplitude between the non-target and target trials. Unlike the *combined task and rest* analyses, not all of the 5 components shown for each frequency band were significant after multiple comparisons corrections were made. Instead, non-significant components were also included if they exhibited functionally interesting correlation maps.

##### Theta band (4–8 Hz)

In the theta band (4–8 Hz), significant task-related activity (p_corrected_ < 0.05) was found in the right motor cortex. In addition, two significant but separate frontal components were identified, similar to the theta band frontal components found in the *combined task and rest* analysis and shown in Fig. 5. A component that corresponded to activity in the SPL was also found but this component did not survive correction for multiple comparisons (p_corrected_ = 0.21). Another non-significant component was found (p_corrected_ = 0.27), corresponding to activity in the lateral visual areas.

##### Alpha/low beta band (8–20 Hz)

In the alpha/low beta band (8–20 Hz), two significant task-related components were found (p_corrected_ < 0.05) that corresponded to the bilateral insula and medial cerebellum. Three non-significant components were also identified that resembled the left lateral frontoparietal network (p_corrected_ = 0.12), the default mode network, (p_corrected_ = 0.14) and the left motor cortex (p_corrected_ = 0.16).

##### High beta/low gamma band (20–40 Hz)

In the high beta/low gamma bands (20–40 Hz), two significant task-related components (p_corrected_ < 0.05), corresponding to activity in the visual cortex and bilateral hippocampi, were found. An additional near-significant component was found (p_corrected_ = 0.055) which described activity in the premotor areas. Two non-significant components were found that corresponded to activity in the SPL (p_corrected_ = 0.15) and the left motor cortex (p_corrected_ = 0.22).

## Discussion

This work can be divided into two parts; the first being an investigation into the optimum way to detect functional connectivity robustly in source space MEG data. In order to quantify functional connectivity, the oscillatory amplitude envelope was estimated on beamformed data and down-sampling was used to focus on the low frequency fluctuations in the envelope. An investigation into the optimum level of down-sampling was carried out in order to assess the best level of down-sampling that separates genuine FC from artefactual FC due to cross-talk between source-space voxels arising from the ill-posed nature of MEG source reconstruction.

The second part of this paper took the finding of this optimum down-sampling window and combined it with ICA with the aim of finding interesting functional networks in task-positive MEG data. A general framework for applying ICA and the GLM in combination to source space MEG data was outlined and applied to a *2-back* working memory paradigm in order to elucidate the underlying electrical activity. The ICA was able to extract a range of functionally meaningful components, and the GLM was used to identify those components that showed significant task-related activity that was also orthogonal to eye-blink activity. This coupling of techniques is very effective at efficiently decomposing source space task-positive MEG data into functionally meaningful, task-related networks. This method also overcomes the challenge of multiple comparisons correction in mass univariate statistical analyses (e.g. using the GLM alone) and provides a method for accounting for non-neural artefacts such as eye-blinks.

### Optimisation of down-sampling

The exploration of down-sampling of the oscillatory amplitude envelope yielded several interesting findings. We confirmed that down-sampling of the envelopes of voxel pairs does indeed boost functional connectivity estimates between genuinely connected regions. This is thought to be due to low frequency power fluctuations being strongly indicative of underlying network behaviour. The down-sampling process behaves like a low-pass filter, blocking out the high frequency oscillatory envelope activity associated with local, non-network processes. However, we also demonstrated that down-sampling boosts correlations between time series with artificially introduced envelope correlations. This is because the standard error on the correlation coefficient is proportional to 1/√N (where N is the number of observations). However, because we are using the absolute value of the correlation coefficient, this causes the mean of the correlation coefficient under the null hypothesis to also be proportional to 1/√N. By using non-parametric statistics, we were able to estimate the optimum down-sampling window length (Δ) that maximises the beneficial effects of the low-pass filtering whilst minimising the boost to artificial FC due to the reduction in number of samples. In other words, we explored how best to separate genuine and artificial populations of FC between node pairs. In this study, artificial correlation was introduced via cross-talk between the beamformer weights of different locations. However, this method is equally applicable to other sources of artificial correlation such as those caused by lead field correlations when using minimum norm source reconstruction methods. Note that this method of simulating artefactual connectivity does not account for all processes that generate spurious functional connectivity (including beamformer-projected cardiac artefacts and mains interference); however, these sources of artificial FC tend to be less problematic in functional connectivity measurements.

We explored the separation between genuinely and artificially connected populations as a function of down-sampling window length, frequency and experimental state (i.e. when at rest, during the task and rest blocks or during the task blocks only). A key finding was that the optimum down-sampling window for estimating functional connectivity was between 1 and 4 s. This finding holds true across the frequency range of 0–150 Hz, in all three experimental states, and is in agreement with previous studies (Brookes et al., 2011b, 2011c; Liu et al., 2010).

Another key finding was the frequency dependency of separation between genuine and artificial connectivity. We found a distinct optimum frequency range of 8–20 Hz, which is in agreement with previous findings (Brookes et al., 2011b; de Pasquale et al., 2010; Liu et al., 2010). We also found that the 30–60 Hz range was least optimal for detecting functional connectivity. However, it is critical to note that our analysis does not prove that functional connectivity does not exist in the 30–60 Hz range; nor does it show that genuine functional connectivity cannot be detected in this range. It shows that any functional connectivity scores measured in this frequency range will be difficult to separate from those caused by artificial processes and due care should be taken to validate them. We also found evidence for detectable functional connectivity in the oscillatory envelopes of high gamma oscillations, from 70 to 150 Hz. In this frequency range, we detected envelope correlations that were well separated from those due to artificial processes. This is a potentially exciting finding but we must highlight two important caveats: firstly, there is a much poorer signal-to-noise ratio in high frequency neural oscillations which will affect any source reconstruction and subsequent analyses; secondly, high frequency activity is strongly contaminated by muscle artefacts and any correlations could be caused by shared contamination. These two points notwithstanding, we believe this to be an interesting avenue for future research and these results are in agreement with recently published invasive findings (Ossandon et al., 2011).

The optimisation analysis also showed that the optimum window length of 1–4 s and frequency band of 8–20 Hz is largely independent of the experimental state, which was either resting-state data, the task and rest blocks of the *2-back* experiment, or the task blocks in isolation. This suggests that the spontaneous low frequency power oscillations associated with resting-state functional connectivity are still present in task-positive data but are additionally modulated by the experimental timing.

The method outlined in this paper relied on a set of pairs of functionally connected voxels to be known a priori. Ideally, a method which does not rely on such information would have been preferable. However, because of the degree of overlap between the distributions of genuinely and artificially connected FC scores, there was no way to separate the two populations (such as the fitting of a mixture model) without knowing which pair-wise correlation scores belong to the connected and unconnected groups. Furthermore, we note that this optimisation does not use any knowledge of the task timings, thereby avoiding any danger of artificially inflating the significance of task-related components.

### Validation of ICA on task-positive data

The correlation maps derived from the components for the *combined task and rest ICA* applied to the theta band show excellent correspondence both with the traditional GLM analysis and with previous findings (Brookes et al., 2011a; Ishii et al., 1999; Jensen and Tesche, 2002; Onton et al., 2005). Most interestingly, in the standard GLM, a single, strong theta band modulation was seen in the frontal cortex. In contrast, the ICA consistently subdivided this activity into two temporally independent components, suggesting that there are two distinct functional processes occurring simultaneously. It should be noted that these two separate networks could be attributed to model order selection: if too many components are modelled then the algorithm may split up a single functional process into multiple independent components; if too few are sought then multiple networks can be merged into a single component (Abou-Elseoud et al., 2010). A full exploration of the effect of ICA model order selection is beyond the scope of this work but should be considered in future applications of this method.

### Elucidation of functional networks

The ICA has yielded several interesting networks all demonstrating significant task‐related activity. Most noteworthy is the default mode network, found in the 8–20 Hz *combined task and rest* ICA, which is generally associated with task-negative processes and is believed to switch off during goal-orientated activities (Fox et al., 2005; Ossandon et al., 2011). Complementing the extraction of the default mode network from this frequency band was the discovery of a bilateral insula component. This component overlaps strongly with the insula nodes of the salience network (Smith et al., 2009). The salience network is postulated to be involved in the switching between brain states and the de-activation of the default mode network during task periods (Sridharan et al., 2008). The extraction of both of these components from a single ICA is extremely interesting and warrants further investigation into the interaction between these two networks. In the 20–40 Hz band, the *combined task and rest blocks* ICA extracted both the default mode network and bilateral somatosensory network, again demonstrating the technique's ability to find functional networks.

Functional networks were also found in the *task blocks only* analyses: the default mode network and the bilateral insular component (which we propose may be part of the salience network) were extracted. However, in this case, only the bilateral insula was found to be significantly related to the presentation of 2-back target trials contrasted against non-target trials. A possible explanation for this is that the salience network is recruited differently in target trials compared with the non-target trials. In contrast, modulation of the default mode network may be insensitive to differences between target and non-target trials. We do not attempt to provide a conclusive answer to this issue but merely wish to highlight another research question that could be investigated using the methods outlined in this study.

Other functional networks found include the lateral visual areas and visual cortex. Both of these visual networks have been found in fMRI analyses of resting-state data using spatial ICA (Smith et al., 2009). However, in this study the lateral visual areas were not significantly related to the task, whereas the medial visual areas were. Future work could investigate whether this corresponds to a difference in the processes being carried out by these networks and their relevance to working memory tasks.

In general, we would emphasise that elucidation of these different networks in MEG data is not currently possible without the use of ICA. In addition, the inclusion of the GLM greatly enhances the technique's ability to determine which networks found by the ICA are involved with the task being analysed.

### *Combined task and rest* vs. *Task blocks only*

The two parallel analyses, ICA applied to the *combined task and rest* blocks of the *2-back* task and ICA applied to the *task blocks only*, generated different results. Many of the most interesting networks were found in both analyses but with different levels of task-related significance. The two types of analysis also yielded some different networks, such as the left motor cortex, which was found in the *task blocks only* analysis. We would expect this because the *2-back* task involves a motor element (the participant was required to press a button with their right hand whenever they detected a *2-back* target).

This shows that ICA can both detect the functional networks that explain the variance in neural activity observed across the task and rest periods (as found by the *combined task and rest* ICA) and the functional networks which explain the neural activity during the WM task alone (as shown by the *task blocks only* ICA). In general, the *task block only* components had much weaker task dependency; only 7 of 15 of the ICs shown (taken from separate ICAs of three frequency bands) were significant (p < 0.05) after correcting for multiple comparisons. This is likely due to the contrast between *2-back* targets and non-targets being much weaker than that between task and rest blocks, allowing for greater inter-subject variability. Another possible explanation is that the higher variability in the task-versus-rest contrast compared with the target-versus-non-target contrast allows the ICA to separate the different components out more robustly. However, it must be noted that the *task blocks only* ICA effectively had half the available data of the *combined task and rest*, potentially leading to a less accurate ICA decomposition and this may account for the weaker statistical results.

Finally, by selecting a down-sampling window length of 0.5 s, we only just retain sensitivity to within-trial oscillations. Limited to four samples per trial, the *task blocks only* independent components will exhibit much greater inter-trial variability and thus higher variance in the GLM regression parameters, potentially explaining the much weaker statistics compared with the combined task and rest analysis (where there are 60 samples per block). A shorter down-sampling window length might reduce this problem but at the cost of being able to separate genuine functional connectivity from artificial connectivity. As the optimisation of down-sampling analysis applied to the *task blocks only* showed, down-sampling improves functional connectivity estimation in the *task blocks only* analysis using window lengths of up to 4 s, even with its shorter event design (see Fig. 3(f)).

The *task blocks only* ICA also showed that a block design experiment is not necessarily required for successful application of ICA. One key caveat is that temporal ICA assumes temporal independence between the different sources. In an evoked response analysis, this assumption may be violated. The inclusion of the rest blocks will assure some degree of temporal independence in *the combined task and rest* analysis. No such guarantee exists for the *task blocks only* ICA. It is not immediately clear when the violation of assumed temporal independence destabilises the ICA (McKeown and Sejnowski, 1998). The results in this study are sufficiently credible that it is unlikely to have occurred here but this is a key issue to consider when applying temporal ICA.

### Localisation of hippocampal activity

An interesting outcome of the ICA when applied to task-positive data was the localisation of activity in the area of the hippocampus. Significant task-related activity centred on the hippocampi was found in the 4–8 Hz *combined task and rest* ICA analysis and both the *combined task and rest* and *task blocks only* analyses for the 20–40 Hz band. These components were tested with the mixed-effects GLM and found to be significantly related to the task timing (p_corrected_ < 0.05). Moreover, the GLM included the eye-blink activity as a nuisance regressor thus showing that the hippocampal components were not being driven by eye-blink activity bleeding into the temporal lobes.

There is contention in MEG imaging as to whether hippocampal activity can be detected due to its cytoarchitecture producing weak magnetic fields and its depth within the head leading to a poor signal-to-noise ratio. Quraan et al. (2011) demonstrated via a range of simulations that hippocampal activity can be detected in MEG data with a beamformer and suitable analysis techniques. Their simulations showed that at least 150 trials are needed per subject for a cohort of at least 12 subjects to localise the hippocampi accurately (these conditions are met by this *2-back* paradigm). In addition, Tesche and Karhu (2000) identified hippocampal activity in subjects undergoing a working memory task, specifically in the theta band, whilst Axmacher et al. (2010) linked theta phase with gamma amplitude by directly measuring local field potentials in the hippocampus during a working memory task. Therefore, the theta and gamma frequency bands would be optimal for hippocampus localisation. This is indeed found to be the case with the best results for hippocampus localisation occurring in the 4–8 Hz (theta) band and 20–40 Hz band (which overlaps with the gamma band).

It stands to reason that ICA is well-suited to detect weak subcortical sources as it can separate the weak but temporally structured signal from the unstructured noise that dominates other analysis techniques. Indeed, this study combined a number of features that increased our chances of detecting hippocampal activity. These included the combination of a working memory task (known to cause fluctuations in theta and gamma band hippocampal activity), the beamformer (which is effective at reconstructing activity at a location in the presence of interference from other locations (Sekihara et al., 2002)) and ICA (which can detect underlying weak signals in a noisy mixture). However, with limited spatial resolution we cannot be completely sure whether the activity is coming directly from the hippocampus or from neighbouring (and likely to be functionally related) cortical areas.

### Coupling of ICA and the GLM

A common critique of the ICA method is that components of interest and components relating to artefacts are generated in a random order. Often, arbitrary heuristics are used to separate out these two groups, potentially biasing the analysis to prior expectations. By using a GLM, each component is assigned a statistical value of task-related dependency allowing functionally interesting components to be separated from noise components, thus reducing this bias.

As well as the GLM enhancing ICA, the converse is also true. Statistical analyses must correct for multiple comparisons (Nichols and Hayasaka, 2003; Woolrich et al., 2009). In its simplest form, multiple comparison correction involves reducing statistical confidence values to account for the number of tests being carried out: the more tests that are required, the more severe the corrections. Multiple comparisons correction is a particularly challenging issue in MEG analysis as the data can have up to six dimensions (3 × space plus time and frequency and subjects) over which statistical tests are performed. In some cases, standard mass univariate statistical methods cannot cope with this. In our method, ICA reduces the data so efficiently that, for a single subject, the number of tests is vastly reduced to (at most) the number of components (around 20, instead of the number of voxels which is of the order of 10,000 in normal GLM analyses). This reduction in the number of statistical tests is not limited to analyses that use the GLM but can in fact be applied to any framework that employs a mass univariate statistical test. Furthermore, the ICA results can be used to identify important brain areas for “region of interest” analyses, a technique that was not employed in this study but that could easily be developed in future studies.

## Conclusion

In summary, two methods were presented in this study. The first is a non-parametric statistical technique for assessing the optimum time-scale to detect functional connectivity in the oscillatory envelope of source space MEG data. This analysis has provided a suitable guide for any study looking to analyse functional connectivity via envelope correlation methods in MEG data. In this study, we have only focussed on the envelope of oscillations whilst ignoring the phase. However, we believe that analysis of the envelope and phase of neural oscillations together will yield the richest understanding of network behaviour in the brain.

We have also presented a novel analysis framework for MEG data, coupling ICA with the GLM, that can overcome many of the limitations of either approach when applied in isolation. The method has been successfully validated on the well-characterised *2-back* working memory paradigm. The task-positive ICA approach both confirms previous findings and yields novel insights into the networks that are involved in working memory, showing that it has great potential as an exploratory analysis technique in MEG.

## Figures and Tables

**Table 1:** The coordinates of the functionally connected nodes used in the optimisation of down-sampling. In the DMN, all four nodes are assumed to be connected to each other. 10 pair-wise correlation scores are estimated for each of the 12 subjects.

Network	MNI coordinates (mm)		
Right LFPN	50	− 62	26
26	32	26
Left LFPN	− 50	− 62	40
− 26	32	40
DMN	42	− 62	36
− 42	− 62	36
0	− 52	28
0	60	4
Motor cortices	41	− 25	49
− 41	− 25	49
Hippocampi	30	− 14	− 16
− 30	− 14	− 16
